# LIN-12/Notch Regulates GABA Signaling at the *Caenorhabditis elegans* Neuromuscular Junction

**DOI:** 10.1534/g3.118.200202

**Published:** 2018-06-27

**Authors:** Altar Sorkaç, Michael A. DiIorio, Patrick J. O’Hern, Saba N. Baskoylu, Hannah K. Graham, Anne C. Hart

**Affiliations:** Department of Neuroscience, Brown University, Providence, Rhode Island, 02912

**Keywords:** LIN-12, Notch, GBB-2, *C. elegans*, neuromuscular junction

## Abstract

The role of Notch signaling in cell-fate decisions has been studied extensively; however, this pathway is also active in adult tissues, including the nervous system. Notch signaling modulates a wide range of behaviors and processes of the nervous system in the nematode *Caenorhabditis elegans*, but there is no evidence for Notch signaling directly altering synaptic strength. Here, we demonstrate Notch-mediated regulation of synaptic activity at the *C. elegans* neuromuscular junction (NMJ). For this, we used aldicarb, an inhibitor of the enzyme acetylcholinesterase, and assessed paralysis rates of animals with altered Notch signaling. Notch receptors LIN-12 and GLP-1 are required for normal NMJ function; they regulate NMJ activity in an opposing fashion. Complete loss of LIN-12 skews the excitation/inhibition balance at the NMJ toward increased activity, whereas partial loss of GLP-1 has the opposite effect. Specific Notch ligands and co-ligands are also required for proper NMJ function. The role of LIN-12 is independent of cell-fate decisions; manipulation of LIN-12 signaling through RNAi knockdown or overexpression of the co-ligand OSM-11 after development alters NMJ activity. We demonstrate that LIN-12 modulates GABA signaling in this paradigm, as loss of GABA signaling suppresses LIN-12 gain-of-function defects. Further analysis, *in vivo* and *in silico*, suggests that LIN-12 may modulate transcription of the GABA_B_ receptor GBB-2. Our findings confirm a non-developmental role for the LIN-12/Notch receptor in regulating synaptic signaling and identify the GABA_B_ receptor GBB-2 as a potential Notch transcriptional target in the *C. elegans* nervous system.

Conserved roles for the Notch signaling pathway in development have been extensively studied across metazoan model organisms (Artavanis-Tsakonas *et al.* 1999; Fortini 2009). Recent studies reveal roles for Notch signaling in non-embryonic tissues, including neurons (Marathe and Alberi 2015). Among the roles of Notch signaling in the nervous system, regulation of synaptic strength, and synaptic activity-dependent behaviors such as learning and memory are prominent. In *Drosophila*, Notch pathway proteins have been implicated in long-term memory (Ge *et al.* 2004; Presente *et al.* 2004; Song *et al.* 2009), alcohol memory (Kaun *et al.* 2011) and olfactory adaptation (Lieber *et al.* 2011). Studies in mice show that the role of Notch in modulating synaptic strength is conserved across species (Costa *et al.* 2003; Wang *et al.* 2004; Conboy *et al.* 2007; Dahlhaus *et al.* 2008; Alberi *et al.* 2011; Brai *et al.* 2014) and the effect of Notch signaling differs from one tissue to another. For instance, increased Notch signaling in the primary visual cortex impairs long-term potentiation (LTP) (Dahlhaus *et al.* 2008), whereas in the hippocampus it favors LTP (Costa *et al.* 2003; Alberi *et al.* 2011). Liu *et al.* recently reported that the transcription factor RBP-Jκ, which is the downstream activator in Notch signaling, regulates γ-aminobutyric acid (GABA) signaling in the adult hippocampus (Liu *et al.* 2014). Despite this, the transcriptional targets of the Notch pathway have not been elucidated in behavioral contexts.

In the nematode *Caenorhabditis elegans*, there are two Notch receptors and they were initially identified for their roles in development. LIN-12 was identified in screens for specification of vulval fates (Greenwald *et al.* 1983; Ferguson and Horvitz 1985); whereas GLP-1 was first characterized as a regulator of mitosis in the germline (Austin and Kimble 1987). In canonical Notch signaling, Notch receptor undergoes a series of cleavages upon activation by ligands. This results in the release of the intracellular domain of Notch that subsequently translocates into the nucleus and associates with the transcription factor LAG-1 and other co-factors to initiate expression of target genes (reviewed in (Kopan and Ilagan 2009)). Studies in *C. elegans* identified a total of ten DSL (Delta/Serrate/LAG-2) proteins as canonical Notch ligands (Lambie and Kimble 1991; Henderson *et al.* 1994; Mango *et al.* 1994; Chen and Greenwald 2004) and five DOS (Delta/OSM-11) motif proteins as possible co-ligands (Komatsu *et al.* 2008). Other than roles in development, canonical Notch signaling in *C. elegans* regulates many behaviors, such as spontaneous reversals in adults (Chao *et al.* 2005), chemotaxis, and sleep (Singh *et al.* 2011).

Here, we assess the role of Notch signaling at the *C. elegans* neuromuscular junction (NMJ), the most studied synapse in this organism. The NMJ consists of three main elements: the muscle, cholinergic motor neurons that excite the muscles (reviewed in (Rand 2007)) and GABAergic motor neurons that inhibit them (reviewed in (Schuske *et al.* 2004)). Acetylcholine (ACh) in the synaptic cleft is broken down by the enzyme acetylcholinesterase, terminating the excitation. An inhibitor of this enzyme, aldicarb, has been extensively used by the *C. elegans* community to identify genes important for proper synaptic signaling at the NMJ (Nonet *et al.* 1993; Rand 2007). Inhibition of the NMJ activity by GABA signaling is not mediated only through ionotropic GABA_A_ receptors expressed in the muscle. Metabotropic GABA_B_ receptors, GBB-1 and GBB-2 are expressed in cholinergic neurons. They are thought to sense spillover GABA in periods of high activity and act through inhibitory G-protein Gα_o_ to decrease ACh release. Since GABA release is dependent on ACh signaling, inhibition via GABA_B_ receptors creates a negative feedback loop on cholinergic neurons (Dittman and Kaplan 2008; Schultheis *et al.* 2011).

In this study, we report that Notch signaling alters response to aldicarb. We identify components of the Notch signaling pathway necessary for proper synaptic transmission. Using genetic and pharmacological manipulations, we elucidate a role for LIN-12 in the regulation of GABAergic signaling at the NMJ via the GBB-2 GABA_B_ receptor, a likely Notch transcriptional target.

## Materials and Methods

### C. elegans strains

Strains used for this study are listed in Table S1. Unless the allele was temperature sensitive all strains were reared at 25° under standard conditions, except for levamisole assays for which the animals were reared at 20°. For all experiments young adult animals were used. All animals carrying the *lin-12(n137)* mutation were kept as heterozygotes over the *unc-32(e189)* variant, singled to homozygose the *n137* mutation, and the progeny of homozygous animals were tested for all assays. *lin-12(n941)* variant was kept on the homozygous lethal *qC1[dpy-19(e1259) glp-1(q339) qIs26]* balancer chromosome; homozygous *lin-12(n941)* mutants were used for experiments. *lag-1(om13)* animals were tested within one month of thawing from frozen stock. All experiments were performed at room temperature (22°), unless the alleles were temperature sensitive. Temperature sensitive mutants and their respective controls were reared at the permissive temperature, switched to the restrictive temperature in the first larval stage and assayed as young adults. These animals were kept at the restrictive temperature in between aldicarb time points.

### Pharmacological assays

Aldicarb (Sigma 33386) and levamisole (Sigma L9756) assays were performed as previously described (Sorkaç *et al.* 2016). Nematode growth medium (NGM) plates with 1mM aldicarb or 0.4mM levamisole were prepared the day before the assay. On the day of the assay, plates were seeded with 30μL *E. coli* strain OP50 and left to dry for 30min. Young adult animals were transferred onto plates and scored for paralysis every hour for 6-8 hr. Paralysis was defined as absence of both movement and pharyngeal pumping upon prodding with a platinum wire. For RNAi experiments, 1mM aldicarb plates were prepared with 1mM ampicillin, 1mM tetracycline and 1mM isopropyl β-D-1-thiogalactopyranoside (IPTG). These plates were seeded with 30μL *E. coli* strain Ht115 carrying either the empty RNAi vector or the RNAi vector of the corresponding gene, according to the RNAi feeding strain on which the animals were kept before the aldicarb assay. Log-rank p-value, pairwise over strata, was calculated for significance using the Kaplan-Meier estimator from IBM SPSS Statistics 22.

Muscimol (Sigma G019) assays were performed similarly to what was done in (Han *et al.* 2015). 1mM muscimol plates were prepared the same way as aldicarb plates. One control and one experimental young adult were transferred onto 1mM muscimol plates. The first photo was taken at t = 0min. The second photo was taken at t = 60min at the same magnification level. Images were analyzed on ImageJ. The midline of each animal was traced with the segmented line tool, and the length of the midline trace was calculated in pixels. The lengths at t = 0min and t = 60min per single animal were used to calculate percent elongation after 1h of 1mM muscimol treatment. Student’s *t*-test was performed to calculate significance.

### Heat-shock experiments

Heat-shock (hs) animals were reared at 15°, switched to 20° the night before the assay. The day of the assay, a 75 min heat-shock treatment was applied at 35° to young adults, followed by 1h of recovery at 20°. Mock heat-shock (no hs) controls were treated the same way without the 75 min heat-shock at 35°.

### RNAi experiments

RNAi plates were prepared by adding ampicillin, tetracycline and IPTG to 1mM final concentrations into the standard NGM mixture. Plates were seeded with 10X concentrated *E. coli* strain HT115 carrying corresponding RNAi vectors or the control vector. After drying, gravid adults were egg prepped onto the seeded plates using a 2.5% bleach+1N NaOH solution. For *osm-11* RNAi aldicarb assays, hatched wild type animals were transferred to corresponding RNAi feeding bacteria in the late second larval stage. Other RNAi experiments were performed using a neuronal RNAi sensitive strain KP3948 *eri-1(mg366)* IV; *lin-15B(n744)* X.

For *apx-1*, animals were assayed on aldicarb as first generation, RNAi-treated young adults. For *dsl-7* and *dos-3* aldicarb trials, second generation, RNAi-treated young adults were used. *osm-11* and *apx-1* RNAi clone was obtained from the Vidal library and *dos-3* clone from the Ahringer library. *dsl-7* RNAi clone was made using *dsl-7* genomic sequence flanked by primers 5′-ATGCTGACTTTATGGTCTTTACTGTTG-3′ and 5′-TTACGACTGTGAATTTAGTCTAACAGG-3′.

### Puncta analysis

Young adult animals carrying the *juIs1[unc-25p*::*snb-1*::*GFP + lin-15(+)]* IV transgene in a wild type or *lin-12(n137)* background were paralyzed in 30mg.mL^-1^ 2,3-Butanedione monoxime (BDM, Sigma B0753) in M9 and mounted on 2% agar pads. Z-stack (distance between images: 0.2μm) images of SNB-1::GFP puncta were taken from the ventral nerve cord between motor neurons VD10 and VD11, using the Zeiss AxioImager ApoTome microscope at 100X magnification. Images formed by merging 3 layers were analyzed using the ‘punctaanalyser” MATLAB program from (Kim *et al.* 2008). Results from control and experimental animals were tested for significance using Student’s *t*-test.

### Data availability

For wild type responses in Table 1 the raw data are available at GSA FigShare. All data necessary for confirming conclusions of this article are represented in figures and tables. All the strains used in this study, and raw data for all experiments are available upon request. Supplemental material available at Figshare: https://doi.org/10.25387/g3.6480419.

**Table 1:** Aldicarb response of ligand or co-ligand mutant animals. Loss of OSM-7, OSM-11 or DSL-3 phenocopies LIN-12 loss, whereas decreased LAG-2, DOS-1 or DSL-6 results in resistance to aldicarb similarly to decreased *glp-1* function. n/a indicates “not available”. At least 10 animals tested for *dos-2*, *arg-1*, *dsl-2*, *dsl-4* and *dsl-5*. For RNAi experiments, three independent trials were conducted using the neuronal RNAi-sensitive strain *eri-1(mg366)*; *lin-15B(n744)*, with a total of at least 30 animals for each knocked-down gene. For non-wild type responses, aldicarb-induced paralysis rates are in Figure S2. For wild type responses the raw data are available in supplements

Gene	Function	Allele	Effect on function	Aldicarb Response
*osm-7*	DOS co-ligand	*tm2256*	null	hypersensitive
*osm-11*	DOS co-ligand	*rt142*	null	hypersensitive
*dos-1*	DOS co-ligand	*ok2398*	null	resistant
*dos-2*	DOS co-ligand	*tm4515*	putative null	wild type
*dos-3*	DOS co-ligand	n/a	RNAi knockdown	wild type
*lag-2*	DSL ligand	*q420*	loss of function	resistant
*arg-1*	DSL ligand	*ok3127*	putative null	wild type
*apx-1*	DSL ligand	n/a	RNAi knockdown	wild type
*dsl-1*	DSL ligand	*ok810*	null	wild type
*dsl-2*	DSL ligand	*tm1805*	putative null	wild type
*dsl-3*	DSL ligand	*ok3411*	putative null	hypersensitive
*dsl-4*	DSL ligand	*ok1020*	putative null	wild type
*dsl-5*	DSL ligand	*ok588*	putative null	wild type
*dsl-6*	DSL ligand	*ok2265*	putative null	resistant
*dsl-7*	DSL ligand	n/a	RNAi knockdown	wild type

## Results and Discussion

### LIN-12 and GLP-1 Notch receptors regulate signaling at the *C. elegans* neuromuscular junction

**Figure 1:**
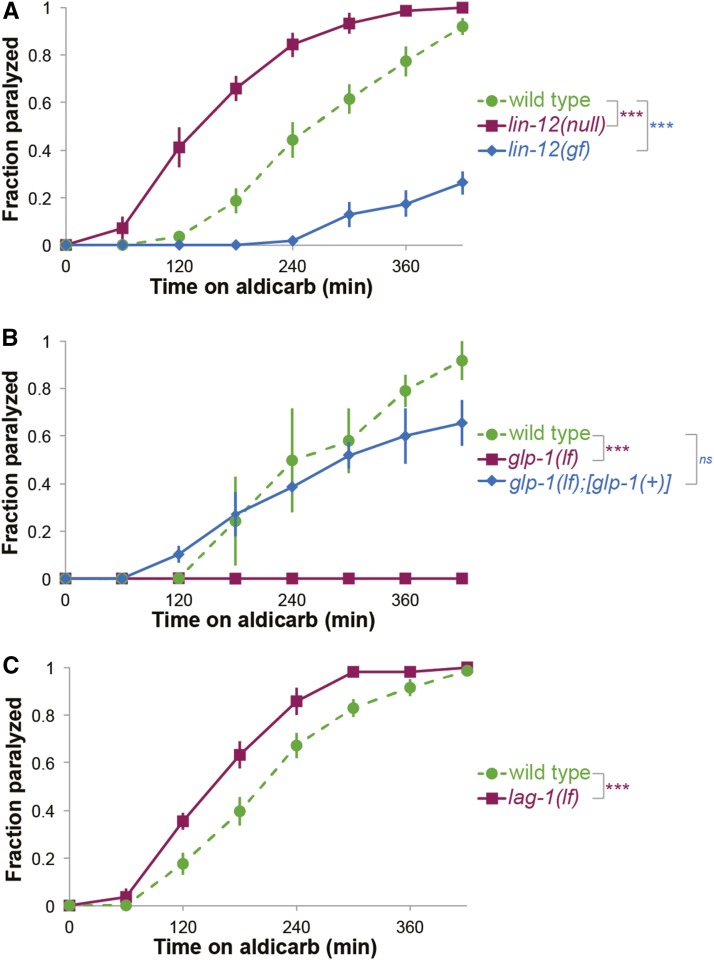
Notch signaling is required for proper activity at the NMJ. (A) Time course of paralysis for *lin-12(n941null)* and *lin-12(n137gf)* mutants in response to aldicarb. Decreased LIN-12 signaling accelerates paralysis upon aldicarb treatment whereas (B) decreased GLP-1 signaling results in strong resistance to aldicarb induced paralysis. *glp-1(q231lf)* is rescued by transgenic *glp-1* expression. (C) Partial loss of the CSL transcription factor LAG-1, *lag-1(om13lf)*, confers hypersensitivity to 1mM aldicarb. (at least three independent trials for each experiment, n > 30 for all genotypes, error bars represent SEM *: log-rank p-value < 0.05, **: log-rank p-value < 0.01, ***: log-rank p-value < 0.001, ns: no significant difference. For (B) and (C) all animals were reared at 15°C and switched to the restrictive temperature of 25°C in the first larval stage.)

Aldicarb causes paralysis of wild type animals at a specific rate and significant deviations from this rate indicate altered signaling at the NMJ. To investigate the regulation of NMJ function by Notch signaling, we examined paralysis rates of Notch receptor loss-of-function (lf) and gain-of-function (gf) mutant animals on 1mM aldicarb. Animals that completely lack LIN-12/Notch signaling, *lin-12(n941null)*, paralyzed faster than wild type control animals. Consistent with this result, *lin-12(n137)* strong gf mutants were resistant to aldicarb, paralyzing more slowly (Figure 1A). The paralysis rate of animals carrying the weaker, cold sensitive gf mutation *lin-12*(*n137n460)* did not differ from control wild type animals (Figure S1A). And, animals carrying the weaker lf mutation *lin-12(q269)* trended toward mild aldicarb hypersensitivity, but not significantly (Figure S1B). Taken together, these results indicate that large changes in LIN-12 receptor signaling impact NMJ activity.

Decreased function of GLP-1, the other *C. elegans* Notch receptor, had the opposite impact on NMJ function. *glp-1(q231)* temperature sensitive lf mutant animals reared at the restrictive temperature failed to paralyze after 7 hr on 1mM aldicarb (Figure 1B). Another lf mutation, *glp-1(q224)*, caused a less dramatic resistance to aldicarb (Figure S1C). The resistance of *glp-1(q231)* animals was rescued by the introduction of an integrated multi-copy transgene that expresses normal GLP-1 protein under the control of the *glp-1* promoter (*tnIs39*, (Sallee *et al.* 2015)) (Figure 1B). Moderate changes in GLP-1 signaling, in *glp-1*(*bn18)* lf or *glp-1(ar202)* gf animals, did not alter aldicarb response (Figure S1D). We conclude that activity of both LIN-12 and GLP-1 Notch receptors impacts response to aldicarb and NMJ function.

In canonical Notch signaling, the intracellular domain of the activated Notch receptor associates with the CSL (mammalian CBF1/RBP-Jκ, *Drosophila* Suppressor of Hairless, *C. elegans* LAG-1) transcription factor. Having observed opposing aldicarb response defects after perturbation of the two *C. elegans* Notch receptor genes, we next examined the aldicarb response of animals with impaired LAG-1 activity to determine if this canonical downstream target is involved. If *lag-1(lf)* animals exhibit an increased rate of paralysis, then LIN-12 signaling is likely functions through LAG-1 in this paradigm. Alternatively, if *lag-1(lf)* animals are resistant, then GLP-1 likely acts via the canonical LAG-1 pathway. Since complete loss of LAG-1 function results in lethality, we used *lag-1(om13)* temperature-sensitive, partial lf animals. They were hypersensitive to aldicarb when reared at the restrictive temperature; this defect was reminiscent of defects seen when LIN-12 function was lost (Figure 1C). This result supports the hypothesis that LIN-12 signals through the canonical pathway, but we cannot draw any conclusions about downstream targets of GLP-1 signaling.

### Identification of pertinent ligands

*C. elegans* Notch receptors are activated by DSL ligands and DOS-motif proteins are thought to act as co-ligands. Given the altered responses of receptor mutant animals to aldicarb, we investigated whether loss of various ligands or co-ligands altered aldicarb paralysis rates (Figure S2). Complete or partial loss-of-function alleles were available for eight out of ten DSL ligands and for four out of five DOS co-ligands. *apx-1* loss is lethal and no alleles were available for two other genes; their impact on NMJ function was examined by RNA interference (RNAi). For this we knocked-down gene function by feeding animals bacteria expressing double-stranded RNA for the corresponding gene. For RNAi studies, we used a *C. elegans* strain whose neurons are hypersensitive to RNAi by feeding. The results are summarized in Table 1.

### OSM-11 is necessary and sufficient for modulation of the NMJ

**Figure 2:**
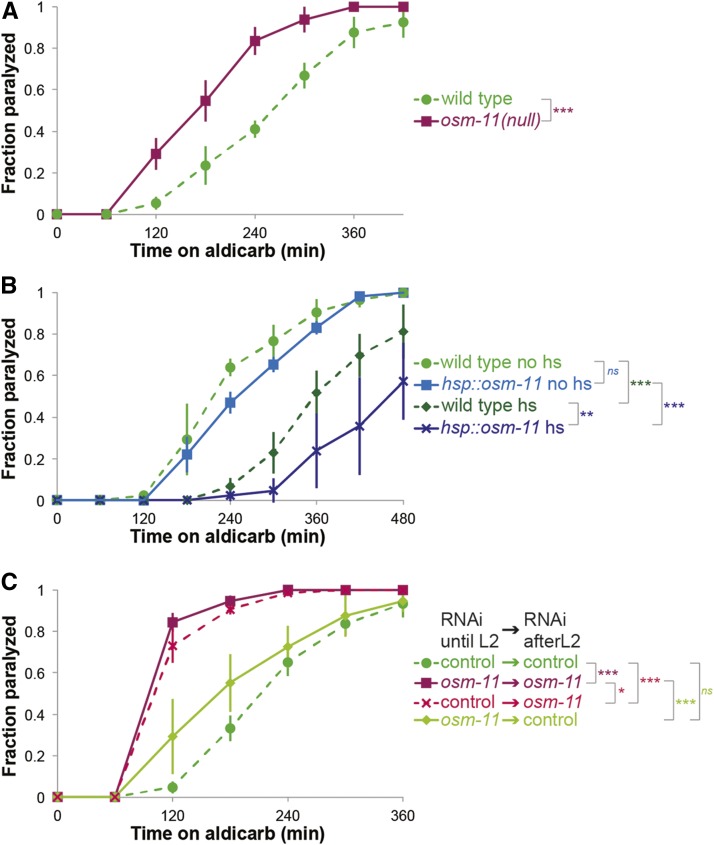
Altering OSM-11 DOS co-ligand levels affect the paralysis rate on aldicarb. (A) Response of *osm-11(rt142null)* animals to aldicarb. Animals that lack the DOS co-ligand OSM-11 are hypersensitive to aldicarb, similar to *lin-12(null)* animals. (B) Overexpression of OSM-11 in adults by heat-shock causes resistance to aldicarb compared to heat-shocked wild type controls. (C) *osm-11* knockdown by feeding RNAi recapitulates aldicarb hypersensitivity seen in *osm-11(rt142null)* animals. Knockdown of *osm-11* starting from late L2 is enough to cause increased hypersensitivity to aldicarb in adults and restoring *osm-11* expression after late L2 stage reverses this hypersensitivity. (hs: heat-shock, no hs: mock heat-shock, at least three independent trials for each experiment, n > 30 for all conditions, error bars represent SEM *: log-rank p-value < 0.05, **: log-rank p-value < 0.01, ***: log-rank p-value < 0.001, ns: no significant difference.)

For the remainder of this study, we focused on the LIN-12 Notch receptor. The secreted DOS co-ligand *osm-11* is epistatic to *lin-12* in determination of vulval cell fates (Komatsu *et al.* 2008). Loss of the secreted DOS co-ligand OSM-11 leads to aldicarb hypersensitivity, as does LIN-12 loss (Figure 2A). It seemed likely that OSM-11 activates the LIN-12 Notch receptor in this paradigm. If so, then increased OSM-11 might induce aldicarb resistance, reminiscent of *lin-12(n137gf)* mutant animals. We used a transgenic line in which expression of *osm-11* is under the control of a heat-shock promoter (Singh *et al.* 2011) to determine if increasing OSM-11 in adult animals would slow aldicarb paralysis rates in otherwise wild type animals. We noted that, even without the transgene, heat-shock slightly increased the aldicarb resistance of wild type animals. But, the impact of OSM-11 over-expression was much more dramatic; elevating OSM-11 in adult animals resulted in even slower paralysis (Figure 2B). These results suggest that increasing the OSM-11 activity in adult animals was sufficient to alter the aldicarb response and that the role of Notch signaling in this paradigm is independent of cell-fate decisions. To confirm these results, we examined the impact of *osm-11* knockdown by feeding RNAi on aldicarb response.

Reminiscent of *osm-11(null)* animals, aldicarb hypersensitivity is seen in wild type animals fed bacteria expressing double stranded *osm-11* RNA (*osm-11*→*osm-11*) For these studies, aldicarb response is compared to control animals reared on empty RNAi vector carrying bacteria (control→control) (Figure 2C). However, given the various roles of Notch signaling in nervous system development, we wanted to rule out cell-fate decisions as a possible cause of the aldicarb phenotypes we observe. Since the identities of body wall muscles and almost all *C. elegans* nervous system including cholinergic and GABAergic neurons at the NMJ are determined by the end of the second larval stage (Sulston 1976; Sulston and Horvitz 1977), we examined the impact of knocking-down *osm-11* from late second larval (L2) stage onwards. If these animals become hypersensitive to aldicarb, cell-fate decisions in the nervous system are likely irrelevant in this paradigm. We found that L2 animals switched from control RNAi to *osm-11* RNAi (control→*osm-11*) exhibited faster paralysis on aldicarb as young adults, recapitulating *osm-11* lf defects (Figure 2C). Conversely, animals reared on *osm-11* RNAi and switched to control RNAi plates as late L2s (*osm-11*→control) exhibited paralysis rates comparable to animals fed with control RNAi throughout larval development (Figure 2C). We conclude that restoring OSM-11 expression after neuronal cell-fate decisions have been made is sufficient to rescue the aldicarb response to wild type levels. Next, we aimed to identify which neurotransmitter signaling system is modulated by LIN-12 signaling in the developed nervous system.

### LIN-12 Notch receptor modulates GABA signaling

Aldicarb directly impairs acetylcholinesterase and alters cholinergic signaling at the NMJ. But, the *C. elegans* NMJ is also directly influenced by GABA neurotransmission. GABA mutant animals have accelerated paralysis on aldicarb (Loria *et al.* 2004) and Notch pathway signaling alters GABA levels in mouse hippocampus (Liu *et al.* 2014). Hence, we investigated whether the aldicarb defects observed in *lin-12* mutants were due to altered GABA signaling.

**Figure 3:**
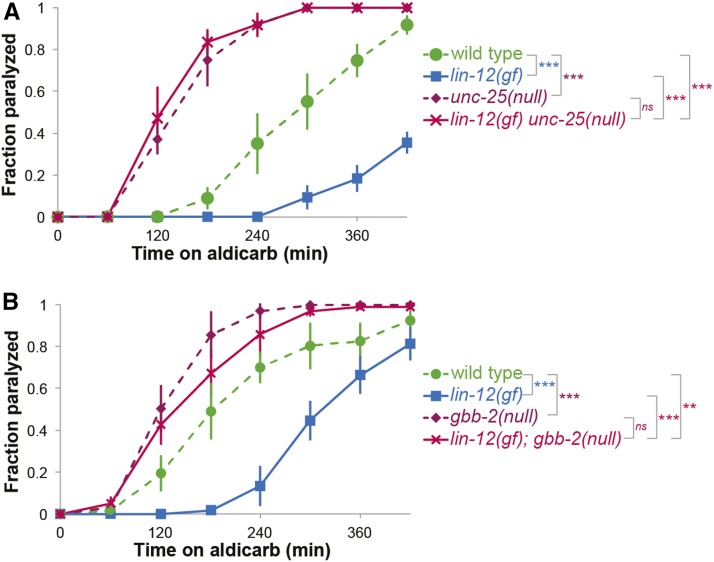
LIN-12 regulates GABA signaling. (A) Paralysis rates of *lin-12(n137gf);unc-25(null)* double mutants on 1mM aldicarb. Loss of GABA production completely suppresses the aldicarb resistance of *lin-12(n137gf)* animals. (B) Paralysis rates of *lin-12(n137gf);gbb-2(null)* double mutants on 1mM aldicarb. Lack of GBB-2 GABA_B_ receptor subunit completely suppresses the aldicarb resistance observed in *lin-12(n137gf)* animals. (at least three independent trials for each experiment, n > 30 for all genotypes, error bars represent SEM *: log-rank p-value < 0.05, **: log-rank p-value < 0.01, ***: log-rank p-value < 0.001, ns: no significant difference.)

Glutamic acid decarboxylase (GAD) converts glutamic acid to GABA at the NMJ. The *C. elegans* GAD ortholog is encoded by the gene *unc-25* and animals that completely lack UNC-25 activity are hypersensitive to aldicarb, due to the decreased muscle inhibition (Loria *et al.* 2004). If the decreased rate of paralysis observed in *lin-12(n137gf)* animals is due to changes in GABA, then loss of *lin-12* likely causes increased GABA signaling. And, completely abolishing GABA production in *lin-12(n137gf)* animals should result in paralysis on aldicarb at a rate comparable to that of *unc-25(e156null)* animals. Consistent with this hypothesis, we found that complete loss of GABA production in *lin-12(n137gf) unc-25(null)* double mutant animals suppressed the *lin-12* gf aldicarb resistance defect; these animals were indistinguishable from *unc-25(null)* single mutants in their aldicarb sensitivity (Figure 3A). It is, therefore, likely that changes in LIN-12 signaling result in altered GABA signaling.

Do mutations in the LIN-12 pathway alter pre- or post-synaptic function? In order to assess this, we tested *osm-11(null)* animals on the acetylcholine agonist levamisole since resistance to levamisole indicated muscle defects. Loss of *osm-11* resulted in hypersensitivity to this drug (Figure S3) Mutants hypersensitive to aldicarb and levamisole can exhibit ACh- or GABA-related pre- and/or post-synaptic defects (Loria *et al.* 2004; Vashlishan *et al.* 2008; Jospin *et al.* 2009). Given the aldicarb hypersensitivity of *osm-11(null)* animals we could not draw conclusions as to where the synaptic function is disrupted in LIN-12 pathway mutants. We, therefore, turned to directly testing GABA release and reception.

Post-synaptically, an increase in number or activity of GABA_A_ receptors could account for aldicarb resistance in *lin-12* gf animals, just as loss of GABA_A_ receptors leads to hypersensitivity (Vashlishan *et al.* 2008). Alternatively, *lin-12(gf)* animals may have presynaptic defects, with increased presynaptic input onto the muscles from GABAergic motor neurons. To discriminate between these scenarios, we examined the response of animals to a GABA_A_ receptor agonist and investigated presynaptic synaptobrevin localization.

The drug muscimol is a GABA_A_ receptor agonist that can cause muscle relaxation in *C. elegans*, which is easily assessed as increased body length (McIntire *et al.* 1993). Animals lacking the postsynaptic GABA_A_ receptor subunit encoded by *unc-49* are resistant to muscimol in this assay, while animals with presynaptic GABA defects are sensitive to muscimol exhibit increased body length when exposed to the drug (McIntire *et al.* 1993). If muscles of *lin-12(n137gf)* animals are more responsive to GABA, muscimol exposure should elongate these animals, compared to wild type animals. However, we found that the muscimol-induced elongation of *lin-12(n137gf)* animals was not significantly different than elongation of the wild type controls (Figure S4A), ruling out a postsynaptic defect due to overactive or more numerous GABA_A_ receptors. Given these results, it is possible that the aldicarb resistance of *lin-12(n137gf)* animals is due to presynaptic defects.

To visualize presynaptic terminals, we examined animals that express *C. elegans* synaptobrevin, SNB-1 tagged with GFP under the control of a GABAergic promoter (Jorgensen *et al.* 1995). Examination of the ventral cord, between motor neurons VD10 and VD11, (Figure S4B) revealed no difference in puncta fluorescence intensity (Figure S4C), volume (Figure S4D) or density (Figure S4E). This result suggests that increased LIN-12 activity does not lead to synaptic vesicle accumulation in GABA motor neurons nor does it increase the size or number of GABAergic synapses.

### GBB-2 is downstream of the LIN-12 Notch receptor in modulating the NMJ function

To define the mechanism through which LIN-12 impacts NMJ function, we looked for potential LIN-12/LAG-1 transcriptional targets. Specifically, we looked for genes 1) that are known to modulate or play an intrinsic role in GABA signaling, 2) whose loss of function results in hypersensitivity to aldicarb and 3) with conserved consensus LAG-1 binding sites (either 5′-YRTGRGAA-3′ or 5′-YGTGDGAA-3′ (Greenwald and Kovall 2013) in putative transcriptional regulatory sequences, based on comparison across four *Caenorhabditis* species: *C. elegans*, *C. briggsae*, *C. remanei* and *C. brenneri*. Only one gene satisfied all of these criteria: *gbb-2*, which encodes a metabotropic glutamate receptor.

At the NMJ, muscles and neurons respond independently to GABA. GABA directly opposes muscle contraction via GABA_A_ receptors expressed by body wall muscle cells. But, GABA also activates GABA_B_ receptors expressed in cholinergic motor neurons and decreases synaptic release via the G_o_ protein alpha subunit GOA-1, thereby indirectly opposing muscle contraction. Loss of either *C. elegans* GABA_B_ receptor, *gbb-1* or *gbb-2*, or loss of *goa-1* results in hypersensitivity to aldicarb, due to increased acetylcholine release by cholinergic motor neurons (Dittman and Kaplan 2008). Genomic sequences near the *C. elegans gbb-2* gene translation initiation site contain two consensus LAG-1 binding sites: one in the third intron and one in the fourth intron. A consensus LAG-1 binding site in the fourth intron is also found in *C. briggsae*, *C. remanei* and *C. brenneri* (Fig S5). Since *gbb-2* is a potential LIN-12/LAG-1 target, we determined if loss of *gbb-2* would suppress the aldicarb resistance of *lin-12(n137gf)* animals. As predicted, the aldicarb response of *lin-12(n137)*; *gbb-2(tm1165null)* double mutant animals was statistically indistinguishable from that of *gbb-2(null)* single mutants (Figure 3B), indicating that *gbb-2* acts downstream of *lin-12* signaling in this paradigm.

The data presented in this article suggests that Notch signaling is required for proper neuromuscular junction signaling in *C. elegans*. Although the role of Notch signaling as a regulator of cell-fate specification has been studied extensively in the field, Notch also alters adult nervous system functioning. In addition to numerous other behaviors and the role Notch plays in muscle arm development in vulval NMJ (Li *et al.* 2013) we present, for the first time in *C. elegans*, that Notch signaling regulates synaptic function.

This is not the first time that the two Notch receptor paralogs have been shown to regulate the same process in the nervous system of *C. elegans*. Loss of LIN-12 or GLP-1 results in an increase in the amount of sleep animals exhibit during the last larval molt, possibly due to the low quality of sleep (Singh *et al.* 2011). The two Notch receptors synergistically affect the response of animals to aversive chemicals (Singh *et al.* 2011). LIN-12 and GLP-1 affect the reversal rate of adult animals in a complex manner ((Chao *et al.* 2005), Hart Lab unpublished results). However, Notch receptors do not always affect the same processes in the nervous system. In 2012, El Bejjani and Hammarlund showed that loss of LIN-12 increased regeneration rates in injured GABA motor neuron processes, whereas decreased GLP-1 signaling had no effect on regeneration (El Bejjani and Hammarlund 2012). In our paradigm, LIN-12 and GLP-1 affect NMJ signaling in opposing manners.

This is particularly interesting since the intracellular domains of GLP-1 and LIN-12, important for canonical Notch signaling, have been shown to be highly homologous (Yochem and Greenwald 1989) and GLP-1 can fully substitute for LIN-12 in vulval cell-fate decisions (Fitzgerald *et al.* 1993). Differential modulation of the NMJ by the two Notch receptors can be explained by these two receptors acting in different tissues and/or at different times. The two receptors might also be altering different communication mechanisms between neurons, namely neurotransmitters and neuropeptides, since neuropeptides have also been shown to alter aldicarb responses (Vashlishan *et al.* 2008; Zhang and Kubiseski 2010; Hu *et al.* 2011; Choi *et al.* 2015). It can further be speculated that one of the receptors act through a non-canonical signaling pathway. Although there is currently no evidence for *C. elegans* Notch receptors to engage in a non-canonical cascade of protein interactions, previous *Drosophila* axon pathfinding studies suggest such an interaction (Crowner *et al.* 2003; Le Gall *et al.* 2008).

In *C. elegans* canonical Notch signaling, intracellular domains of activated Notch receptors associate with the CSL transcription factor LAG-1 to initiate transcription of target genes. Given the fact that partial loss LAG-1 accelerates the paralysis of animals on aldicarb, it is very likely that the LIN-12 Notch receptor acts through the canonical pathway to alter the signaling at the NMJ. The same cannot be said for the GLP-1 receptor. We tried to monitor for genetic interactions between GLP-1 and *C. elegans* orthologs of proteins from the *Drosophila* non-canonical signaling pathway. However, these proteins did not seem to modulate GLP-1 mediated regulation of the NMJ (data not shown).

From our analysis using ligand or co-ligand loss-of-functions, we speculate as to which ligands are acting on which receptors in our paradigm. Since loss of DSL-3, OSM-7 or OSM-11 leads to hypersensitivity to aldicarb, they are likely acting on LIN-12 Notch receptor. LAG-2, DSL-6 and DOS-1 likely activate GLP-1 given that their losses phenocopy *glp-1(lf)* animals. Further studies are required to provide more specific conclusions about the genetic interactions between the ligands and the corresponding receptors.

We chose to manipulate levels of the DOS co-ligand OSM-11 to exclude developmental defects as a basis for the aldicarb sensitivity we observed in *lin-12(null)* animals, as complete loss or overexpression of OSM-11 does not lead to infertility or lethality (Komatsu *et al.* 2008; Singh *et al.* 2011). Overexpression of this secreted co-ligand in adults slowed down paralysis rates on aldicarb. This result has two implications. First of all, it shows that increasing levels of a Notch co-ligand is sufficient to induce resistance to aldicarb. Furthermore, it corroborates the idea that the changes we observed in LIN-12 mutants are not due to developmental defects caused by altered cell-fate decisions. However, overexpression of a secreted protein can have ectopic effects. To overcome this problem, we knocked-down *osm-11* starting at the end of the second larval stage. By this time, animals have mostly completed cell-fate specification in their nervous system. Decreasing levels of OSM-11 in late L2 animals caused hypersensitivity to aldicarb, suggesting that the phenotype is not due to improper cell-fate decisions. Moreover, this phenotype is easily reversible by restoring OSM-11 synthesis. This suggests that absence of the Notch co-ligand OSM-11 during development does not interfere with the regulation of the adult NMJ by Notch signaling.

Further genetic analysis of *lin-12(gf)* animals showed that their resistance to aldicarb was dependent on increased GABAergic signaling; the phenotype is completely suppressed by abolishment of GABA production through loss of UNC-25. The mechanism through which LIN-12 increases GABA signaling involves the GBB-2 GABA_B_ receptor subunit that detects spillover GABA and modulate acetylcholine release from cholinergic motor neurons (Dittman and Kaplan 2008; Schultheis *et al.* 2011). Although our analysis lacks the direct evidence for transcriptional control of *gbb-2* under the control of LIN-12 Notch receptor, the presence of conserved LAG-1 binding sites in the *gbb-2* gene supports regulation. Future studies should focus on the context in which the Notch pathway regulates GABA signaling and the role of the GBB-2 receptor.

## References

[bib1] Alberi L. Liu S. Wang Y. Badie R. Smith-Hicks C., 2011 Activity-induced Notch signaling in neurons requires Arc/Arg3.1 and is essential for synaptic plasticity in hippocampal networks. Neuron 69: 437–444. 10.1016/j.neuron.2011.01.004 21315255 PMC3056341

[bib2] Artavanis-Tsakonas S. Rand M. D. Lake R. J., 1999 Notch signaling: cell fate control and signal integration in development. Science 284: 770–776. 10.1126/science.284.5415.770 10221902

[bib3] Austin J. Kimble J., 1987 *glp-1* is required in the germ line for regulation of the decision between mitosis and meiosis in *C. elegans*. Cell 51: 589–599. 10.1016/0092-8674(87)90128-0 3677168

[bib4] Brai E. Marathe S. Zentilin L. Giacca M. Nimpf J., 2014 Notch1 activity in the olfactory bulb is odour-dependent and contributes to olfactory behaviour. Eur. J. Neurosci. 40: 3436–3449. 10.1111/ejn.12719 25234246

[bib5] Chao M. Y. Larkins-Ford J. Tucey T. M. Hart A. C., 2005 *lin-12* Notch functions in the adult nervous system of *C. elegans*. BMC Neurosci. 6: 45 10.1186/1471-2202-6-45 16011804 PMC1181819

[bib6] Chen N. Greenwald I., 2004 The lateral signal for LIN-12/Notch in *C. elegans* vulval development comprises redundant secreted and transmembrane DSL proteins. Dev. Cell 6: 183–192. 10.1016/S1534-5807(04)00021-8 14960273

[bib7] Choi S. Taylor K. P. Chatzigeorgiou M. Hu Z. Schafer W. R., 2015 Sensory Neurons Arouse *C. elegans* Locomotion via Both Glutamate and Neuropeptide Release. PLoS Genet. 11: e1005359 10.1371/journal.pgen.1005359 26154367 PMC4495980

[bib8] Conboy L. Seymour C. M. Monopoli M. P. O’Sullivan N. C. Murphy K. J., 2007 Notch signalling becomes transiently attenuated during long-term memory consolidation in adult Wistar rats. Neurobiol. Learn. Mem. 88: 342–351. 10.1016/j.nlm.2007.04.006 17543552

[bib9] Costa R. M. Honjo T. Silva A. J., 2003 Learning and memory deficits in Notch mutant mice. Curr. Biol. 13: 1348–1354. 10.1016/S0960-9822(03)00492-5 12906797

[bib10] Crowner D. Le Gall M. Gates M. A. Giniger E., 2003 Notch steers *Drosophila* ISNb motor axons by regulating the Abl signaling pathway. Curr. Biol. 13: 967–972. 10.1016/S0960-9822(03)00325-7 12781136

[bib11] Dahlhaus M. Hermans J. M. Van Woerden L. H. Saiepour M. H. Nakazawa K., 2008 Notch1 signaling in pyramidal neurons regulates synaptic connectivity and experience-dependent modifications of acuity in the visual cortex. J. Neurosci. 28: 10794–10802. 10.1523/JNEUROSCI.1348-08.2008 18945887 PMC6671381

[bib12] Dittman J. S. Kaplan J. M., 2008 Behavioral impact of neurotransmitter-activated G-protein-coupled receptors: muscarinic and GABAB receptors regulate *Caenorhabditis elegans* locomotion. J. Neurosci. 28: 7104–7112. 10.1523/JNEUROSCI.0378-08.2008 18614679 PMC2679701

[bib13] El Bejjani R. Hammarlund M., 2012 Notch signaling inhibits axon regeneration. Neuron 73: 268–278. 10.1016/j.neuron.2011.11.017 22284182 PMC3690129

[bib14] Ferguson E. L. Horvitz H. R., 1985 Identification and characterization of 22 genes that affect the vulval cell lineages of the nematode *Caenorhabditis elegans*. Genetics 110: 17–72. 3996896 10.1093/genetics/110.1.17 PMC1202554

[bib15] Fitzgerald K. Wilkinson H. A. Greenwald I., 1993 *glp-1* can substitute for *lin-12* in specifying cell fate decisions in *Caenorhabditis elegans*. Development 119: 1019–1027. 8306872 10.1242/dev.119.4.1019

[bib16] Fortini M. E., 2009 Notch signaling: the core pathway and its posttranslational regulation. Dev. Cell 16: 633–647. 10.1016/j.devcel.2009.03.010 19460341

[bib17] Ge X. Hannan F. Xie Z. Feng C. Tully T., 2004 Notch signaling in *Drosophila* long-term memory formation. Proc. Natl. Acad. Sci. USA 101: 10172–10176. 10.1073/pnas.0403497101 15220476 PMC454384

[bib18] Greenwald I. Kovall R., 2013 Notch signaling: genetics and structure. WormBook, ed. The C. elegans Research Community, WormBook 1–28. 10.1895/wormbook.1.10.2 PMC5402211 23355521

[bib19] Greenwald I. S. Sternberg P. W. Horvitz H. R., 1983 The *lin-12* locus specifies cell fates in *Caenorhabditis elegans*. Cell 34: 435–444. 10.1016/0092-8674(83)90377-X 6616618

[bib20] Han B. Bellemer A. Koelle M. R., 2015 An Evolutionarily-Conserved Switch in Response to GABA Affects Development and Behavior of the Locomotor Circuit of *Caenorhabditis elegans*. Genetics 199: 1159–1172. 10.1534/genetics.114.173963 25644702 PMC4391577

[bib21] Henderson S. T. Gao D. Lambie E. J. Kimble J., 1994 *lag-2* may encode a signaling ligand for the GLP-1 and LIN-12 receptors of *C. elegans*. Development 120: 2913–2924. 7607081 10.1242/dev.120.10.2913

[bib22] Hu Z. Pym E. C. Babu K. Vashlishan Murray A. B. Kaplan J. M., 2011 A neuropeptide-mediated stretch response links muscle contraction to changes in neurotransmitter release. Neuron 71: 92–102. 10.1016/j.neuron.2011.04.021 21745640 PMC3134788

[bib23] Jorgensen E. M. Hartwieg E. Schuske K. Nonet M. L. Jin Y., 1995 Defective recycling of synaptic vesicles in synaptotagmin mutants of *Caenorhabditis elegans*. Nature 378: 196–199. 10.1038/378196a0 7477324

[bib24] Jospin M. Qi Y. B. Stawicki T. M. Boulin T. Schuske K. R., 2009 A neuronal acetylcholine receptor regulates the balance of muscle excitation and inhibition in *Caenorhabditis elegans*. PLoS Biol. 7: e1000265 10.1371/journal.pbio.1000265 20027209 PMC2787625

[bib25] Kaun K. R. Azanchi R. Maung Z. Hirsh J. Heberlein U., 2011 A Drosophila model for alcohol reward. Nat. Neurosci. 14: 612–619. 10.1038/nn.2805 21499254 PMC4249630

[bib26] Kim J. S. Lilley B. N. Zhang C. Shokat K. M. Sanes J. R., 2008 A chemical-genetic strategy reveals distinct temporal requirements for SAD-1 kinase in neuronal polarization and synapse formation. Neural Dev. 3: 23 10.1186/1749-8104-3-23 18808695 PMC2564922

[bib27] Komatsu H. Chao M. Y. Larkins-Ford J. Corkins M. E. Somers G. A., 2008 OSM-11 facilitates LIN-12 Notch signaling during *Caenorhabditis elegans* vulval development. PLoS Biol. 6: e196 10.1371/journal.pbio.0060196 18700817 PMC2504490

[bib28] Kopan R. Ilagan M. X., 2009 The canonical Notch signaling pathway: unfolding the activation mechanism. Cell 137: 216–233. 10.1016/j.cell.2009.03.045 19379690 PMC2827930

[bib29] Lambie E. J. Kimble J., 1991 Two homologous regulatory genes, *lin-12* and *glp-1*, have overlapping functions. Development 112: 231–240. 1769331 10.1242/dev.112.1.231

[bib30] Le Gall M. De Mattei C. Giniger E., 2008 Molecular separation of two signaling pathways for the receptor, Notch. Dev. Biol. 313: 556–567. 10.1016/j.ydbio.2007.10.030 18062953 PMC2262048

[bib31] Li P. Collins K. M. Koelle M. R. Shen K., 2013 LIN-12/Notch signaling instructs postsynaptic muscle arm development by regulating UNC-40/DCC and MADD-2 in *Caenorhabditis elegans*. eLife 2: e00378 10.7554/eLife.00378 23539368 PMC3601818

[bib32] Lieber T. Kidd S. Struhl G., 2011 DSL-Notch signaling in the *Drosophila* brain in response to olfactory stimulation. Neuron 69: 468–481. 10.1016/j.neuron.2010.12.015 21315258 PMC3216490

[bib33] Liu S. Wang Y. Worley P. F. Mattson M. P. Gaiano N., 2014 The canonical Notch pathway effector RBP-J regulates neuronal plasticity and expression of GABA transporters in hippocampal networks. Hippocampus 25: 670–678. 10.1002/hipo.22402 PMC4412774 25515406

[bib34] Loria P. M. Hodgkin J. Hobert O., 2004 A conserved postsynaptic transmembrane protein affecting neuromuscular signaling in *Caenorhabditis elegans*. J. Neurosci. 24: 2191–2201. 10.1523/JNEUROSCI.5462-03.2004 14999070 PMC6730426

[bib35] Mango S. E. Thorpe C. J. Martin P. R. Chamberlain S. H. Bowerman B., 1994 Two maternal genes, *apx-1* and *pie-1*, are required to distinguish the fates of equivalent blastomeres in the early *Caenorhabditis elegans* embryo. Development 120: 2305–2315. 7925031 10.1242/dev.120.8.2305

[bib36] Marathe S. Alberi L., 2015 Notch in memories: Points to remember. Hippocampus 25: 1481–1488. 10.1002/hipo.22426 25656274

[bib37] McIntire S. L. Jorgensen E. Horvitz H. R., 1993 Genes required for GABA function in *Caenorhabditis elegans*. Nature 364: 334–337. 10.1038/364334a0 8332190

[bib38] Nonet M. L. Grundahl K. Meyer B. J. Rand J. B., 1993 Synaptic function is impaired but not eliminated in *C. elegans* mutants lacking synaptotagmin. Cell 73: 1291–1305. 10.1016/0092-8674(93)90357-V 8391930

[bib39] Presente A. Boyles R. S. Serway C. N. de Belle J. S. Andres A. J., 2004 Notch is required for long-term memory in *Drosophila*. Proc. Natl. Acad. Sci. USA 101: 1764–1768. 10.1073/pnas.0308259100 14752200 PMC341850

[bib40] Rand, J.B. (2007). Acetylcholine. WormBook, 1–21. 10.1895/wormbook.1.131.1 PMC4781110 18050502

[bib41] Sallee M. D. Aydin T. Greenwald I., 2015 Influences of LIN-12/Notch and POP-1/TCF on the Robustness of Ventral Uterine Cell Fate Specification in *Caenorhabditis elegans* Gonadogenesis. G3 (Bethesda) 5: 2775–2782. 10.1534/g3.115.022608 26483009 PMC4683648

[bib42] Schultheis C. Brauner M. Liewald J. F. Gottschalk A., 2011 Optogenetic analysis of GABAB receptor signaling in *Caenorhabditis elegans* motor neurons. J. Neurophysiol. 106: 817–827. 10.1152/jn.00578.2010 21613582 PMC3154801

[bib43] Schuske K. Beg A. A. Jorgensen E. M., 2004 The GABA nervous system in *C. elegans*. Trends Neurosci. 27: 407–414. 10.1016/j.tins.2004.05.005 15219740

[bib44] Singh K. Chao M. Y. Somers G. A. Komatsu H. Corkins M. E., 2011 *C. elegans* Notch signaling regulates adult chemosensory response and larval molting quiescence. Curr. Biol. 21: 825–834. 10.1016/j.cub.2011.04.010 21549604 PMC3100419

[bib45] Song Q. Sun K. Shuai Y. Lin R. You W., 2009 Suppressor of Hairless is required for long-term memory formation in *Drosophila*. J. Neurogenet. 23: 405–411. 10.3109/01677060903096133 19863271

[bib46] Sorkaç A. Alcantara I. C. Hart A. C., 2016 In Vivo Modelling of ATP1A3 G316S-Induced Ataxia in *C. elegans* Using CRISPR/Cas9-Mediated Homologous Recombination Reveals Dominant Loss of Function Defects. PLoS One 11: e0167963 10.1371/journal.pone.0167963 27936181 PMC5148073

[bib47] Sulston J. E., 1976 Post-embryonic development in the ventral cord of *Caenorhabditis elegans*. Philos. Trans. R. Soc. Lond. B Biol. Sci. 275: 287–297. 10.1098/rstb.1976.0084 8804

[bib48] Sulston J. E. Horvitz H. R., 1977 Post-embryonic cell lineages of the nematode, *Caenorhabditis elegans*. Dev. Biol. 56: 110–156. 10.1016/0012-1606(77)90158-0 838129

[bib49] Vashlishan A. B. Madison J. M. Dybbs M. Bai J. Sieburth D., 2008 An RNAi screen identifies genes that regulate GABA synapses. Neuron 58: 346–361. 10.1016/j.neuron.2008.02.019 18466746

[bib50] Wang Y. Chan S. L. Miele L. Yao P. J. Mackes J., 2004 Involvement of Notch signaling in hippocampal synaptic plasticity. Proc. Natl. Acad. Sci. USA 101: 9458–9462. 10.1073/pnas.0308126101 15190179 PMC438998

[bib51] Yochem J. Greenwald I., 1989 *glp-1* and *lin-12*, genes implicated in distinct cell-cell interactions in *C. elegans*, encode similar transmembrane proteins. Cell 58: 553–563. 10.1016/0092-8674(89)90436-4 2758466

[bib52] Zhang Y. Kubiseski T. J., 2010 *Caenorhabditis elegans wsp-1* regulation of synaptic function at the neuromuscular junction. J. Biol. Chem. 285: 23040–23046. 10.1074/jbc.M109.096164 20501656 PMC2906297

